# Clinical impact of diagnostic imaging discrepancy by radiology trainees in an urban teaching hospital emergency department

**DOI:** 10.1186/1865-1380-6-24

**Published:** 2013-07-16

**Authors:** Steven Marc Friedman, Erica Merman, Amit Chopra

**Affiliations:** 1 Emergency Medicine – University Health Network, Faculty of Medicine, University of Toronto, RFE G-S434, 200 Elzaibeth Street, Toronto, Ontario M5G 2C4, Canada

**Keywords:** Discrepant reporting, Radiology, Emergency medicine, Patient safety, Patient outcomes, Mortality, Morbidity, Altered long-term outcome

## Abstract

**Background:**

To characterize clinically significant diagnostic imaging (DI) discrepancies by radiology trainees and the impact on emergency department (ED) patients.

**Methods:**

Consecutive case series methodology over a 6-month period in an urban, tertiary care teaching hospital. Emergency physicians (EPs) were recruited to flag discrepant DI interpretations by radiology trainees that the EP deemed clinically significant. Cases were characterized using chart review and EP interview.

**Results:**

Twenty-eight discrepant reports were identified (representing 0.1% of 18,185 images interpreted). The mean time between provisional discrepant diagnosis (PDDx) and revised diagnosis (RDx) by attending radiology staff was 8.6 h (median 4.8 h, range 1.1-48.4), and 67.9% (n = 19) of the patients had left the ED by time of notification. The most frequently reported PDDx was CT abd/pelvis (32.1%, *n* = 9) and CT head (28.6%, *n* = 8). The impact of RDx was deemed major in 57.1% (*n* = 16) for reasons including altered admitting status (32.1%, *n* = 9), immediate subspecialty referral (*n* = 16, 57.1%), impact on management (25%, *n* = 7), and surgical management (21.4%, *n* = 6). EPs reported likely perceived impact of PDDx as resulting in increased pain (17. 9%, *n* = 5), morbidity (10.7%, *n* = 3), and prolonged hospitalization (25%, *n* = 7), but not altered long-term outcome or mortality.

**Conclusions:**

Relatively few clinically important discrepant reads were reported. Revised diagnosis (RDx) was associated with major clinical impact in 57.1% of reports, but few patients experienced increased morbidity, and none increased mortality. The importance of expedient communication of discrepant reports by staff radiologists is stressed, as is EP verification of patient contact information prior to discharge.

## Background

Patients who present to an urban teaching hospital emergency department (ED) often undergo a diagnostic imaging study (CT, ultrasound, etc.) that is performed after-hours or on weekends. Studies performed after hours are typically first read by a radiology resident, and the emergency physician (EP) may render a treatment plan and disposition based on this ‘preliminary’ diagnostic interpretation. However, the final interpretation by the staff radiologist, minutes to days later, sometimes reveals clinically important discrepancies from the initial resident interpretation and results in a revised interpretation that may alter patient management and outcome.

Thus, a patient may present to the ED with a headache, have a CT read as normal by the radiology resident, and be discharged by the emergency physician with a diagnosis of a benign headache, only for the final read up to 36 h later to reveal an intracranial hemorrhage. Similarly, a CT scan ordered for abdominal pain may be read as normal by the radiology trainee and the patient sent home by the EP with a diagnosis of gastroenteritis, only for the scan to be ultimately revised by the staff radiologist as appendicitis. Such patients must then be contacted to return to the ED, often urgently, for further investigation and treatment. The purpose of this study was to characterize a consecutive case series of clinically significant diagnostic imaging (DI) discrepancies in an urban teaching hospital ED and the impact they have on ED patients.

The current literature on discrepant reporting seeks mostly to quantify the percentage of misreads that occurs between radiology residents and staff radiologists as well as to characterize the types of scans that are most often misinterpreted. In reviewing previous studies, Stevens et al. reported a “wide variation in reported discrepancy rates between preliminary reports provided by the on-call radiology resident and the final report from the subspecialty attending radiologist, ranging from 3% - 29.9%,” and note that 0.5% - 5.0% were recorded as “major” events [1]. Cooper et al. found that the rate of discrepant reporting between radiology residents and staff was low, reporting 3.3% for minor discrepancies and 1% for major discrepancies, with this rate decreasing slightly with each additional year of residency training and more common for body CT than other imaging modalities. (The decision to classify as minor or major was done by the radiologist, not EM staff) [2]. Maloney et al. reviewed 2,255 preliminary interpretations by radiologists using a validated scoring system (RADPEER) and judged 1.29% to have potentially clinically significant interpretations. The authors report “CT imaging generated a higher percentage of discrepancies that were predicted to be clinically significant than plain film radiography, as well as a higher percentage of discrepancies that resulted in immediate changes in management, but the incidence of each remained low overall” [3]. Ruutiainen et al. reported that in a review of 45,608 preliminary interpretations by radiology residents at an academic institution, 0.89% had major discrepancies [4]. Discrepancy rates varied by modality (greatest with CT imaging) and year of training.

Radiology resident image interpretation in academic centers has been generally viewed as meeting accepted quality control standards. For example, Blane et al. report that radiology residents handled off-hours cases with a radiology-detected error rate below the inter-observer error rate between ABR-certified radiologists [5]. Eng et al. performed a comparison of emergency medicine physicians with radiologist, radiology residents with faculty, and film with digital display. The authors estimated in conclusion that radiology residents in-house, covering the ED and interpreting film, provided a performance improvement similar to that of teleradiology coverage by a faculty radiologist. The authors report “we determined that physician specialty, training level, and image display method has significant associations for the accuracy of interpretation of emergency department radiographs” [6].

We hypothesize that while the incidence of clinically significant misreads in the emergency department is low, there is a discrete frequency of misreads (typically false negatives) that may be characterized as adverse events that have clinical impact on the patient, including progression of illness, delay of definitive care, prolonged pain, prolonged hospitalization, and increased morbidity and mortality. The purpose of this study was to characterize these events in the two EDs of a downtown teaching hospital.

## Methods

In a 6-month prospective cohort study using a consecutive case series methodology, we identified and tracked cases of discrepant radiology reports in the two emergency departments of University Health Network, Toronto. This is a two-site, merged, urban, tertiary care teaching hospital that sees approximately 100,000 ED visits per year, of which approximately 17% are admitted to the hospital (2012 data). In our hospital, protocol directs that radiology resident misreads are brought to the attention of the emergency physician by telephone or electronically by the staff radiologist (and by telephone if critical), after reviewing and correcting a report by the on-call radiology resident from the evening before. During the study period, 40 emergency physicians (EPs) were regularly directed to register in the trial cases that they regarded as constituting potentially clinically significant discrepant reads. EP notifications included biweekly email reminders and verbal announcements at monthly departmental meetings. The emergency physician was directed to enter the discrepancy into the study (by recording the EP name, date and patient identifiers in a study binder) if they felt that the misread was clinically significant. “Clinically significant” was defined to EPs as instances where *“if you had to call the patient back, and/or alter management, and/or were concerned that there was or could have been significant impact for the patient or for you.”* When asked for further clarification, EPs were advised that final revised reports advising delayed outpatient follow-up, i.e., a repeat CXR in 6–8 weeks to better distinguish a potential lucency from an artifact, would not merit inclusion in the study.

Reported cases were assessed using (1) chart review and (2) physician interview. After providing informed consent, the EP who logged the case for the study (and who also initiated patient contact and potentially altered management following the revised DI diagnosis) underwent a scripted telephone interview with one of the researchers (E.M.) to capture clinical and operational correlates of the discrepant report. This interview served as the primary source for characterizing the EP’s perceived clinical impact on the patient. Further clinical, operational, and social correlates were abstracted using a review of the patient’s handwritten ED chart, electronic medical record, and radiology information system (RIS). Social correlates abstracted from the chart review include age, gender, need for a translator, and housing status prior to admission. Chart abstraction and telephone interviews were performed by a researcher (E.M.) who was unblinded but not familiar with the individual emergency physicians. The study was piloted for 4 weeks prior to study launch, with consequent minor adjustments in the script and questions for clarity. In order to minimize potential for the Hawthorne effect, the radiology department was not made aware of this study. The protocol was approved by the hospital research ethics board.

Outcome measures included: (1) perceived clinical impact for the patient, including pain, morbidity, delayed care, altered clinical outcome, prolonged hospitalization, and anxiety/distress; (2) social correlates, including patient age, fluency in English, affiliation with a primary care physician, and housing status prior to admission; (3) operational correlates of the incident, including the type of imaging requested, regional anatomy, and delay to informing the emergency physician. Data analysis was performed using Excel and SAS.

## Results

ED staff logged 28 discrepant reports over the 6-month period (15 April–15 October 2011). The mean time between the provisional discrepant diagnosis (PDDx) and revised diagnosis (RDx) was 8.6 h (median 4.8 h, range 1.1-48.4 h). Approximately two thirds of patients (67.9%, *n* = 19) had left the ED at the time of notification of the EP with the revised diagnosis.

### Patient characteristics

The median patient age was 53 (*n* = 28, range 15–97 years); all (*n* = 28) provided an emergency contact on their chart, and 71.4% (*n* = 20) listed a family physician. Almost all patients who experienced discrepant reads were living at home prior to hospital admission (*n* = 27, 96%), and most spoke English as their first language (*n* = 21, 75%). Only one patient of 28 required a translator. All patients could be reached by telephone by the EP doing follow-up of the final revised report.

Errors in the provisional diagnosis were characterized according to the type of scan, region of anatomy, and type of error (false negative, false positive, or incidental finding). The most frequently reported PDDxs were CTabd/pelvis followed by CT head. However, CT extremity had the highest percentage of reported significant misreads relative to other regions of anatomy (see Table 1). The majority of the discrepant reads were false negatives (*n* = 23, 82%; see Table 2).

**Table 1:** **Characterizing diagnostic errors: CT scans** and **ultrasounds (*n *= 28)**

**Region of anatomy scanned**	**Six-month ED usage**	**Significant discrepancy reported**	**Percentage**	**95 % CI**
**CT head**	2,798	8	0.29 %	(0.12%, 0.56%)
**CT chest**	404	3	0.74 %	(0.15%, 2.15%)
**CT abdo/pelvis**	1,359	9	0.67 %	(0.30%,1.25%)
**CT spine/neck**	255	1	0.39 %	(0.01%, 2.17%)
**CT extremity**	72	2	2.8 %	(0.34%, 9.68%)
**US abdo/pelvis**	1,354	2	0.15 %	(0.02%, 0.53%)
**X-Ray**	11,943	3	0.03 %	(0.01%, 0.07%)
**Total:**	18,185	28	0.10 %	(0.10%, 0.22%)

**Table 2:** **Characterizing diagnostic errors by error type (*n *= 28)**

		
**False negative**	*n* = 23, 82%	(63.11%, 93.94%)
**False positive**	*n* = 4, 14%	(4.03%, 32.67%)
**Incidental finding**	*n* = 1, 4%	(0.09%, 18.35%)

### Rating events by emergency department chart review

The impact of the RDx was categorized as major in 57.1% (*n* = 16) of cases by a rating emergency physician (SF). Criteria for designating impact as major were based on those previously utilized by Stevens et al. and included one or more of: altered admitting status (i.e., from outpatient to admitted), referral for immediate subspecialty consultation, significant impact on medical management, and surgical management [1] (see Table 3).

**Table 3:** **Major clinical impacts associated with revised diagnosis (*n *= 28 cases)**

		
Altered admitting status	*n* = 9, 32.1%	95% CI (15.9%, 52.4%)
Immediate subspecialty referral	*n* = 16, 57.1%	95% CI (37.2%, 75.5%)
Impact on management	*n* = 7, 25%	95% CI (10.7%, 44.9%)
Surgical management	*n* = 6, 21.4%	95% CI (8.3%, 41.0%)

Minor clinical impacts associated with the RDx were identified in 89.3%, (*n* = 25) of cases for reasons including need for follow-up imaging (*n* = 10, 36%), need for outpatient specialty referral (*n* =15, 54%), and change in treatment not adversely affected by a 24-h delay in diagnosis (*n* =19, 68%).

### Interview of emergency physician reporting discrepant diagnosis

Each emergency physician who logged a clinically significant discrepant case was subsequently interviewed regarding his or her perceptions of the impact of the respective discrepant DI diagnosis. EPs reported increased patient pain (17. 9%, *n* = 5 cases), morbidity (10.7%, *n* = 3), and prolonged hospitalization (25%, *n* = 7) for the patient, but not altered long-term outcome or mortality.

## Discussion

In our study, only a very small proportion of diagnostic imaging studies performed on ED patients were subsequently logged by ED physicians as clinically significant misreads. The clinical impact of revised diagnosis (RDx) was deemed to be major in 57.1% of reported cases. However, few patients experienced increased morbidity, and none was felt to have experienced increased mortality or altered long-term outcome. Patients discharged home with discrepant reads were not significantly socially compromised, in that they tended not to be elderly, had an address and phone number where they could be reached, and spoke English.

Stevens et al. analyzed discordance rates between preliminary radiology resident reports and final reports from attending radiologists on 2,830 cross-sectional imaging studies requested by ED staff after hours. Using similar scoring criteria and ED chart review, the authors reported discrepancies in 2.0% of studies overall, with 1.6% of overall studies having a significant discordance and 0.43% requiring an immediate change in management [1]. Other studies have reported major discrepancy rates (between preliminary radiology resident read and final staff radiologist read) ranging from 0.5% to 5.0% [7-10].

Clinically significant misreads between radiology residents and staff radiologists typically consist of false negatives [11,12], meaning that a potentially clinically important finding might be overlooked. Relatively few studies have looked at the clinical impact of these misinterpretations. Carney et al. observed a 1.0% rate of major discrepancy and 5.4% rate of minor discrepancy on body CT scans between initial and final interpretation. The authors reported that discrepant reads resulted in changes in patient management but did not lead to increased patient morbidity or mortality [7]. Chung et al. reported a similar rate of misreads resulting in changes in patient management [13]. Lal et al. reviewed neuroradiological CT scans and reported a potentially serious change in patient outcome in only 0.08% of cases [8]. Ruchman et al. reported the discrepancy rate between radiology residents and staff to be 24%, with 7% of these misreads having some negative impact on patient care, but none having a major negative impact on the patient [9]. The type of scan most commonly misread was a contrast-enhanced CT of the abdomen and pelvis [10].

To contextualize these findings, it is important to note that the bulk of research in this area has been completed by radiologists, does not involve ED chart or physician interview, and may not reflect the emergency department interpretation of what constitutes a clinically important discrepancy or patient impact.

### Optimizing care in the ED

Protocolized follow-up is an effective way to ensure that emergency physicians receive notification of any discrepant readings between the radiology resident and staff radiologist. Many academic institutions mandate that the attending radiologist flag all discrepancies on the Radiology Information System (RIS) to facilitate follow-up by the emergency physician. Protocols should be in place that ensure minimal delay between resident interpretation and final radiologist read of diagnostic imaging and also ensure immediate direct and real-time communication between the radiologist and emergency physician in the case of clinically important change in DI interpretation. In our institution, these policies are in effect, but were noted not to have been appropriately followed in several of the reported cases of significant discrepancy involving delayed notification of the emergency physician. We believe that real-time and direct (i.e., telephone) communication between the attending radiologist and emergency physician regarding significant discrepancies – while more time-consuming for the radiologist than indirect methods such as email communication or RIS flagging – leads to better patient care.

The phenomenon of patients being sent home prior to EP and patient notification of a revised diagnostic imaging interpretation increases the potential for an adverse patient outcome. We suggest that EPs, as a policy, advise patients who are admitted to the ED and later discharged “after hours” that there is a possibility that they might be called within the next 24–36 h for follow-up and that EPs verify patient contact information prior to ED discharge. The combination of a significant discrepancy noted in the revised diagnosis of an ED patient after discharge and incorrect contact information in the hospital chart could magnify the potential for an avoidable catastrophic outcome.

The issue of radiology discrepancies from resident radiologist physicians (as compared to attending radiologists) has been addressed in many settings in the US by the requirement for 24-h availability of attending radiologists – whether by an in-house radiologist, teleradiology by on-call radiologists, or intercontinental “outsourcing” for after-hours diagnostic imaging interpretation. The necessity for this and implications for quality and radiologist lifestyle are actively discussed with divergent opinions in the radiology community [5,9,14-17].

This study corresponds with the start of a quality improvement initiative on the part of the radiology department to implement expedited staff radiologist final interpretation of provisional reads by radiology trainees. A turnaround-time goal was established by the radiology departments of our hospital and affiliated teaching hospitals in 2011 that the 90th percentile of turnaround times should be less than 4 h during the day and less than 6 h overnight. The new initiative implemented on-site, out-of–hours staff radiologist coverage to provide final reports for all emergency department and impatient cross-sectional imaging, and all ED radiographs. Between April 2010 and the end of July 2011 – prior to implementation of the initiatives – 2% of daytime ultrasound imaging studies met the 4-h turnaround time target and 25% of overnight studies met the 9-h target. From the time of plan implementation in August 2011 to the spring of 2013, these improved to 68% and 79%, respectively. For computed tomography scans, the respective improvements were from 17% to 76% and from 55% to 89% [18]. The relatively low rates of reported clinically significant discrepant reports in our study may be related in part to the impact of this QI project.

### Limitations

The purpose of this study was not to capture all discrepant reports, but to capture the most important misreads from the EP’s perspective using a new approach. This study was dependent upon recruitment by busy emergency physicians and subject to underreporting. Secondary correlation with the radiology information system to see if all discrepancies were captured was not performed.

Selection by emergency physicians (or by radiologists, as in previous studies) employs a potentially biased interpretation of what constitutes a clinically important diagnostic imaging misread or important patient impact (such as increased pain or morbidity). EPs were repeatedly given a standardized working definition of what constituted a “clinically significant” discrepancy for the purpose of this study (see Methods); however, there was no follow-up to see if there was agreement among EPs when using this definition. Clinical impact on the patient (pain, morbidity, etc.) was based on EP interview and is potentially subjective. Correlation with inpatient charts in the case of admitted patients and long-term follow-up to further explore the impact on morbidity and long-term outcome might have provided correlation of emergency physician impressions.

Chart abstraction and review for clinical impact by a single rater introduce potential for bias. Results may be dependent on the patient population studied and institutional radiology department practices, and may not be generalizable to other institutions. A larger sample size would have allowed for multivariate analysis that might identify characteristics of “high-risk” scans that would benefit from real-time interpretation by attending radiologists.

The radiology resident error rate has been shown to diminish with the level of training [2-4,6,9,19]. We did not attempt to correlate discrepant reads with the level of resident training.

There are others sources of discrepant diagnostic imaging interpretation in the ED – such as ultrasounds now being performed by EPs that are not over-read by radiologists. EP misreads were beyond the scope of this methodology, but also merit study.

## Conclusions

Our study aimed to characterize clinically significant diagnostic imaging (DI) discrepancies by radiology trainees and the impact on emergency department (ED) patients. Relatively few clinically important discrepant reads were reported; however, of those that did occur, most patients were discharged from the hospital before they had been notified of the discrepancy. Although the impact of revised diagnosis (RDx) was deemed to have a major clinical impact in 57.1% of reports, few patients experienced increased morbidity, and none were perceived to have experienced increased mortality or altered long-term outcome at the time of EP follow-up. The importance of expedient, direct communication by radiologists to EPs when noting significant diagnostic imaging discrepancies was reinforced, as was EP verification of accurate contact information prior to discharge. We encourage radiology departments to incorporate emergency department collaboration in quality control audits for diagnostic accuracy and adverse event discovery.

## Abbreviations

CT: Computed tomography; US: Ultrasound; DI: Diagnostic imaging; ED: Emergency department; EP: Emergency physician; PDDX: Provisional discrepant diagnosis; RDx: Revised diagnosis; DI: Diagnostic imaging.

## Competing interests

The authors declare that they have no competing interests.

## Authors’ contributions

Author contribution to this project was as follows: project supervision, conception and design: SF; piloting and refinement of methodology: SF, EM; acquisition of data: EM; data analysis: SF, EM; drafting and revision of manuscript: SF, EM; final approval of the version to be published: SF. All authors read and approved the final manuscript.
